# Rapamycin Inhibits ALDH Activity, Resistance to Oxidative Stress, and Metastatic Potential in Murine Osteosarcoma Cells

**DOI:** 10.1155/2013/480713

**Published:** 2013-02-14

**Authors:** Xiaodong Mu, Christian Isaac, Trevor Schott, Johnny Huard, Kurt Weiss

**Affiliations:** ^1^Stem Cell Research Center, University of Pittsburgh School of Medicine, Bridgeside Point II, 450 Technology Drive, Pittsburgh, PA 15219, USA; ^2^Department of Orthopaedic Surgery, University of Pittsburgh School of Medicine, Bridgeside Point II, 450 Technology Drive, Pittsburgh, PA 15219, USA

## Abstract

Osteosarcoma (OS) is the most common primary malignancy of bone. Mortality is determined by the presence of metastatic disease, but little is known regarding the biochemical events that drive metastases. Two murine OS cell lines, K7M2 and K12, are related but differ significantly in their metastatic potentials: K7M2 is highly metastatic whereas K12 displays much less metastatic potential. Using this experimental system, the mammalian target of rapamycin (mTOR) pathway has been implicated in OS metastasis. We also discovered that aldehyde dehydrogenase (ALDH, a stem cell marker) activity is higher in K7M2 cells than K12 cells. Rapamycin treatment reduces the expression and enzymatic activity of ALDH in K7M2 cells. ALDH inhibition renders these cells more susceptible to apoptotic death when exposed to oxidative stress. Furthermore, rapamycin treatment reduces bone morphogenetic protein-2 (BMP2) and vascular endothelial growth factor (VEGF) gene expression and inhibits K7M2 proliferation, migration, and invasion *in vitro*. Inhibition of ALDH with disulfiram correlated with decreased mTOR expression and activity. In conclusion, we provide evidence for interaction between mTOR activity, ALDH activity, and metastatic potential in murine OS cells. Our work suggests that mTOR and ALDH are therapeutic targets for the treatment and prevention of OS metastasis.

## 1. Introduction

Osteosarcoma (OS), the most common primary malignancy of bone, usually occurs in the long bones during childhood and adolescence at sites of rapid bone turnover [1–3]. Despite pre- and postoperative chemotherapy and wide surgical resection of the tumor, overall survival for patients without radiographically detectable metastases is only 65–70% [1, 2, 4, 5, 8]. The prognosis for patients with detectable metastases at the time of diagnosis is particularly poor, ranging from 15 to 30% [1, 7, 8]. It is thus the presence of pulmonary metastatic disease that ultimately determines OS mortality [9]. However, little is known about the biochemical signaling pathways that drive the progression of metastases and the molecular biology of OS remains poorly understood. As a result, we have yet to develop therapeutic strategies that specifically target metastatic disease.

 The mammalian target of rapamycin (mTOR) is a highly conserved serine/threonine kinase, and as its name implies, mTOR activity is specifically inhibited by the drug rapamycin [10–13]. Rapamycin is an antimicrobial agent produced by *Streptomyces hygroscopius* that also exhibits potent immunosuppressive and antitumor properties, likely due to its ability to arrest the cell cycle in G1-phase [14]. mTOR signaling regulates a number of critical cellular processes including cellular growth, metabolism, and aging via an extraordinarily complex intercellular signaling network [15, 16]. Dysregulation of this mTOR signaling network can participate in a variety of human disease processes including cancer [17]. 

In mammals, mTOR associates with the proteins Raptor or Rictor to form mTOR complexes 1 and 2 (mTORC1 and 2), respectively. mTORC1 activity is sensitive to rapamycin, whereas mTORC2 is not [18, 19]. The best characterized substrates of mTORC1 are p70 ribosomal protein S6 kinase (S6 K1) and the eukaryotic initiation factor 4E-binding protein 1 (4E-BP1), through which mTOR activity can regulate protein synthesis and cell growth [17]. A role for rapamycin-sensitive and rapamycin-insensitive mTOR signaling in cell motility and cancer metastasis is evolving but our current understanding is limited [14]. It is, however, widely recognized that mTOR signaling plays a critical role in protein synthesis, cell proliferation, growth, and survival [10, 20–22]. Dysregulated mTOR signaling is found in a variety of human cancers including hematologic, lung, breast, liver, pancreas, renal, skin, and gastrointestinal tract neoplasms [17]. In addition, it was recently discovered that mTOR signaling is activated in human osteosarcoma and correlates with surgical stage, metastasis, and disease-free survival [23]. The primary goal of this study was to investigate the role of mTOR signaling in OS metastasis and mTOR inhibition with rapamycin. 

K7M2 and K12 are related murine OS cell populations derived from the same spontaneously-occurring OS in a Balb-C mouse. K7M2 cells are highly metastatic to the lungs and were clonally derived from the much less metastatic K12 cells [24]. K7M2 and K12 cells are thus very similar genetically but differ significantly in their metastatic potentials. As such, they represent excellent tools for determining critical biochemical pathways in OS metastasis. It has been reported that mTOR signaling activity is enhanced in K7M2 cells compared to K12 cells [25]. Here we report that mTOR signaling in K7M2 cells is associated with higher aldehyde dehydrogenase (ALDH, a cancer stem cell marker) activity, increased resistance to oxidative stress, increased proliferation, migration, and invasion, and higher bone morphogenetic protein (BMP2) and vascular endothelial growth factor (VEGF) expression than in the less metastatic K12 cells. All of these metastatic phenotypes were reversed with rapamycin treatment. Interestingly, we also report that ALDH inhibition with disulfiram is correlated with decreased mTOR activity and causes morphological alterations to K7M2 cells. This study provides evidence that the mTOR pathway promotes ALDH activity and metastatic potential in OS cells. We conclude that mTOR and ALDH are potential therapeutic targets in the treatment and prevention of OS metastasis. 

## 2. Materials and Methods

### 2.1. Cell Culture and Rapamycin Treatment

K7M2 cells and K12 cells were cultured with proliferation medium (PM; DMEM with 10% FBS and 5% penicillin and streptomycin). For mTORC1 inhibition of K7M2 cells, rapamycin (Sigma) was dissolved in DMSO (10 mM) and diluted 1 : 1000 in proliferation medium to a working concentration of 10 *μ*M. K7M2 cells were seeded in 12-well plastic plates at 5,000 cells per well, and 1 mL treatment medium containing rapamycin was added to each well. 1 mL of medium containing the same amount of DMSO served as control treatment. Treatment medium was refreshed each day and cells were treated for 2 to 4 days.

### 2.2. Fluorescence-Activated Cell Sorting (FACS) Analysis of ALDH Activity and Sorting of Cells

The Aldelfluor Kit (STEMCELL Technologies) was used to determine ALDH enzymatic activity. Cultured K7M2 cells and K12 cells, with or without rapamycin treatment (10 *μ*M for 48 hrs), were resuspended in Aldefluor buffer (1 × 10^6^ cells/mL) and incubated at 37°C according to the manufacturer's instructions. Cells were washed in Aldefluor buffer and maintained in 4°C throughout the cell sorting process. ALDH activity was assessed using the FL1 channel of a BD FACSAria Cell Sorting System and FACSDiva software (version 6.1.2; Becton, Dickinson and Company, San Jose, CA). Collected cells were sorted with fluorescence-activated cell sorting (FACS), according to their fluorescence intensity, which corresponds to their ALDH activity levels, as well as low side scatter (SCC^lo^). ALDH-high cells and ALDH-low cells were separately harvested and cultured.

### 2.3. Cell Proliferation Assay

K7M2 and K12 cells were plated at 1000 cells per well in a 12-well plate and cultured in PM. A time-lapsed microscopic live-cell imaging (LCI) system (Automated Cell, Inc.) was used to take images of cells per each field of view at 15 minute intervals for 4 days. The approximate population doubling time (PDT) as determined as follows: 2^*n*^ = cell number at harvest time/cell number initially plated; “*n*” refers to the number of doublings during the period of cell culture (96 hrs), thus PDT = 96 hrs/*n*. 

### 2.4. Cell Survival Assay after Exposure to Oxidative Stress

The antioxidant capacities of K7M2 (unsorted, ALDH-high, and ALDH-low fraction) and K12 cells cultured in 12-well plastic plates were compared by exposure to oxidative stress (250 *μ*M H_2_O_2_ in PM) for 6 hrs. Also, to test the role of mTOR inhibition on the antioxidant capacity of K7M2 cells, K7M2 cells were pretreated with rapamycin (10 *μ*M) for 48 hrs prior to exposure to oxidative stress (0, 250, or 500 *μ*M H_2_O_2_ in PM) conditions for 6 hours. Propidium iodide (PI) was added to the medium (1 *μ*g/mL) and apoptotic cells were identified with positive PI staining. 

### 2.5. *In Vitro* Single Cell Migration Assay

An automated time-lapsed microscopy system (Biorad) was used to track the single cell migration on plastic surface. Cells were observed at 15 minute increments over 96 hours, the composite images were analyzed, the tracks of migration of 10 preselected single cells were monitored for each cell group, and cell velocities were calculated. 

### 2.6. Cell *In Vitro* Invasion Assay

*In vitro* invasion capacity of K7M2 cells with or without rapamycin treatment, as well as ALDH-high and ALDH-low fractions of untreated K7M2 cells, was assessed using a real-time cell invasion and migration (RT-CIM) assay system (ACEA Biosciences, Inc.), with a 16-well trans-well plate (CIM-plate 16, Roche Diagnostics GmbH). The surface of the wells in the upper chamber was coated with Matrigel (BD BioSciences, Bedford, MA USA) of different concentrations (2.5%, 5%, and 10%). Serum-containing medium (10% FBS) was added to the wells of the lower chamber. Cells (4 × 10^4^ per well) in serum-free medium were seeded in the upper chamber. The migration of the cells through the Matrigel was monitored by the system every 15 minutes for 24 hours. Data analysis was carried out using RTCA Software 1.2 supplied with the instrument.

### 2.7. Semiquantitative Reverse Transcription Polymerase Chain Reaction (RT-PCR)

Total RNA was extracted from the cells using the RNeasy plus mini kit (Qiagen) and cDNA was generated using the iScript cDNA Synthesis kit (Bio-Rad). The sense and antisense primers for RT-PCR and their product sizes are found in Table 1. The cycling parameters used for all reactions were as follows: 94°C for 5 minutes; 30 cycles of the following: denature for 45 seconds at 95°C, anneal for 30 seconds (53°C–56°C), and extend for 45 seconds at 72°C. RT-PCR was performed using a Bio-Rad MyiQ thermal cycler (Bio-Rad). GAPDH served as a control gene, and the expression of target genes was normalized to the expression of GAPDH. Gradient dilution (1 : 1, 1 : 2, and 1 : 4) of RNA samples from different cell groups was compared respectively to verify the quantitative difference of gene expression. RT-PCR analysis was performed using ImageJ software (version 1.32j, National Institutes of Health, Bethesda, MD) where the integrated density (product of the area and the mean gray value) of bands was calculated. All molecular bands were normalized to GAPDH.

### 2.8. Actin Staining

Organization of actin in ALDH-high, ALDH-low, and disulfiram treated cells was assessed using the phalloidin conjugated with Alexa Fluor 488 (Invitrogen). Populations of ALDH-high and ALDH-low cells were plated on to 12 well plates (50,000 cells/plate) and grown overnight in proliferation medium. The following day, the plates were washed twice with PBS, fixed in 3.7% formaldehyde solution for 10 minutes at room temperature, and washed two more times with PBS. The cells were then permeabilized in 0.1% Trition X-100 for 20 minutes and washed again with PBS. For each well a staining solution of 5 *μ*L of methanolic stock solution phalloidin with 200 *μ*L PBS and 1% BSA was added. The staining solution was kept in the wells for 20 minutes, and then the wells were washed again with PBS. This made the actin appear green under a fluorescence microscope. The nucleus was stained by adding 300 *μ*L of 300 nM DAPI to wells for 5 minutes and then rising twice with PBS. This made the nucleus appear blue under a fluorescence microscope. 

### 2.9. Disulfiram Treatment

Disulfiram is an ALDH inhibitor that has been used to treat alcoholism by blocking the enzymatic conversion of ethanol to acetic acid. In this study it was used to determine the effect of blocking ALDH in K7M2 cells vis-à-vis mTOR activity and cell morphology. ALDH-high and ALDH-low cells were plated and left to grow for approximately 2 hours before adding the disulfiram. A concentration of 250 nM was found to be the high nontoxic concentration able to be used on the cells.

### 2.10. mTOR Immunostaining

In order to determine the relative expression of mTOR in disulfiram-treated versus untreated cells, the cells were incubated with p-4E-BP1 (a rabbit antibody to mTOR). After a period of 2 hours, the cells were rinsed and then incubated with a fluorescent anti-rabbit antibody for one hour. The cells were then treated with DAPI as described earlier in order to visualize the nuclei. Pictures were then taken with a fluorescence microscope.

### 2.11. Statistical Analysis

At least three samples obtained from each subject were pooled for statistical analysis of all results from this study, and the results are expressed as a mean ± SD. The differences between two means were considered to be statistically significant if *P* value was <0.05. A Student's *t*-test was used to determine statistically significant differences between two means.

## 3. Results

### 3.1. Rapamycin Inhibits BMP2, VEGF, and ALDH-1A1 Gene Expression in K7M2 Cells

**Figure 1:**
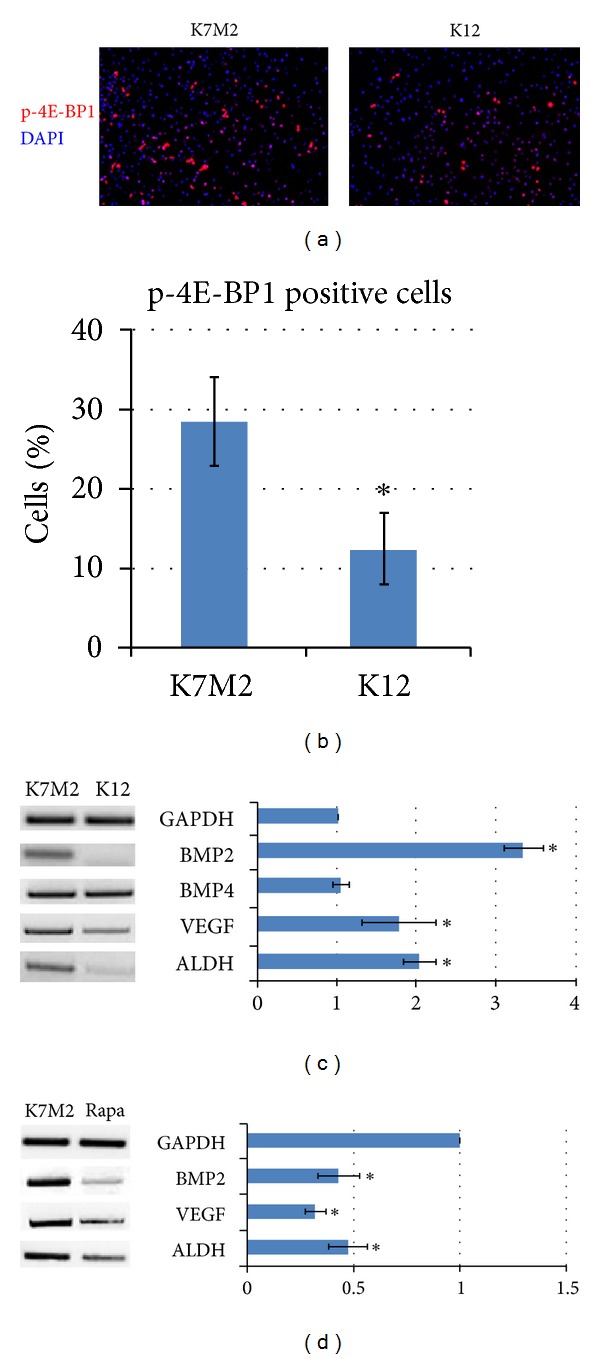
Phospho-4E-BP1 levels and rapamycin-sensitive gene expression in murine OS cells. (a) Immunofluorescence analysis of K7M2 and K12 cells depicting phospho-4E-BP1 (p-4E-BP1) counterstained with DAPI. (b) Quantitative analysis of p-4E-BP1 staining comparing the percentage of positive K7M2 and K12 cells. (c) RT-PCR was performed on cellular RNA extracted from K7M2 and K12 cells in order to quantitate the relative expression of BMP2, BMP4, VEGF, and ALDH-1A1. GAPDH serves as a loading control. (d) RT-PCR was performed on cellular RNA extracted from K7M2 cells treated with rapamycin or DMSO only (K7M2) in order to quantitate the relative expression of BMP2, VEGF, and ALDH-1A1 in the presence of mTORC1 kinase inhibition. Again, GAPDH serves as a loading control. Asterisks (∗) indicate statistically significant differences (*P* < 0.05).

K7M2 cells are known to exhibit greater mTORC1 kinase activity [25]. In order to confirm elevated mTORC1 kinase activity in our K7M2 cells we compared the amount of its phosphorylated substrate, phospho-4E-BP1 (p-4E-BP1), in K7M2 and K12 cells by immunofluorescence using a phosphospecific antibody (anti-phospho-threonine-37/46).  Figure 1(a) shows a representative image of fixed K7M2 and K12 cell populations processed for p-4E-BP1 and DAPI immunofluorescence. Consistent with published results [25], we observed a greater than twofold increase in p-4E-BP1 immunofluorescence in K7M2 cultures compared to K12 cultures (Figure 1(b)), suggestive of elevated mTORC1 kinase activity in the more metastatic K7M2 cells.

 ALDH activity, a “cancer stem cell marker,” is found in a variety of human cancers and has been associated with metastasis, drug resistance, and a poor prognosis [26–30]. In order to investigate the role of mTORC1 activity in the regulation of ALDH in K7M2 cells we first performed reverse transcription polymerase chain reaction (RT-PCR) using RNA extracted from K7M2 and K12 cells treated with rapamycin or DMSO control. We have previously shown that BMP2 and VEGF expressions are upregulated in K7M2 cells compared to K12 cells [31]. Therefore, we analyzed the expression of BMP2, BMP4, and VEGF, in addition to ALDH-1A1 expression. GAPDH served as a loading control.  Figure 1(c) shows the upregulation of these genes, except BMP4, in K7M2 cells compared to K12 cells, in the absence of rapamycin. After the addition of rapamycin to the media, we observed a significant down-regulation of BMP2, VEGF, and ALDH-1A1 mRNA transcripts of K7M2 cells (Figure 1(d)).

### 3.2. Rapamycin Treatment Reduces ALDH Activity and Sensitizes K7M2 Cells to Oxidative Stress

**Figure 2:**
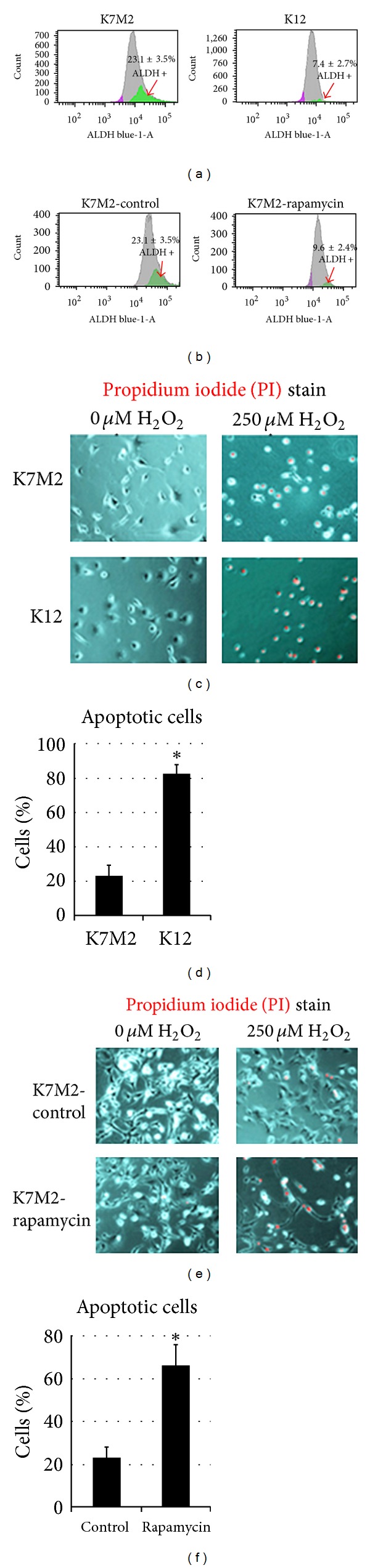
Rapamycin treatment reduces ALDH activity and sensitizes K7M2 cells to oxidative stress. (a) ALDH activity was detected in K7M2 and K12 cells using FACS analysis and the relative amount of cells positive for ALDH is shown for each cell population. (b) K7M2 cells were treated with rapamycin or DMSO only (control) and analyzed by FACS as in (a). (c) K7M2 and K12 cells were treated with or without H_2_O_2_ (250 *μ*M) and apoptosis was detected using PI staining. (d) A quantitative analysis of (c) illustrating the percentage of apoptotic cells after H_2_O_2_ treatment compared to untreated controls. (e) K7M2 cells were treated with or without H_2_O_2_, in the presence or absence (DMSO only control) of rapamycin, and apoptotic cells were detected as in (c). (f) A quantitative analysis of (e) illustrating the percentage of apoptotic cells after H_2_O_2_ compared to untreated controls. Asterisks (∗) indicate statistically significant differences (*P* < 0.05).

In order to further investigate the effect of rapamycin on ALDH we performed fluorescence-associated cell sorting (FACS) analysis to quantitate the percentage of cells with ALDH activity. Consistent with our RT-PCR results, we observed a threefold increase in the percentage of  K7M2 cells with ALDH activity (23.1 ± 3.5% versus 7.4 ± 2.7%) compared to K12 cells (Figure 2(a)). However, after rapamycin treatment, the percentage of K7M2 cells with ALDH activity reduced significantly, compared to DMSO control group, and approached a level of activity more comparable to the less metastatic K12 cells (Figure 2(b)).

After observing that rapamycin effectively reduced ALDH-1A1 expression and ALDH activity in K7M2 cells, we next wanted to investigate the effect of rapamycin treatment on K7M2 resistance to oxidative stress because the activity of ALDH in cancer may function to neutralize oxidative stress and provide chemoresistance [32, 33]. We tested resistance to oxidative stress using H_2_O_2_ treatment. Apoptosis was monitored by nuclear inclusion of propidium iodide (PI).  Figure 2(c) contains representative images of K7M2 cells and K12 cells treated with or without H_2_O_2_. After treatment with H_2_O_2_, the large majority (>85%) of K12 cells underwent apoptosis as indicated by PI inclusion, whereas most of the K7M2 cells maintained viability (>65%) as indicated by nuclear exclusion of PI (Figure 2(d)). Therefore, K7M2 cells are more resistant to oxidative stress from H_2_O_2_ exposure than K12 cells. In order to test if this resistance to H_2_O_2_ was related to mTORC1 or ALDH activity we repeated these experiments on K7M2 cells in the presence or absence of rapamycin, and 0 *μ*M or 250 *μ*M of H_2_O_2_. Representative images are shown in Figure 2(e). We observed an increased sensitivity to H_2_O_2_ with rapamycin treatment as rapamycin caused a threefold increase in apoptosis (Figure 2(f)). Rapamycin alone is not proapoptotic in this assay, as rapamycin in the absence of H_2_O_2_ did not increase the frequency of PI nuclear inclusion in K7M2 cells.

### 3.3. Rapamycin Treatment Reduces K7M2 Cell Proliferation

**Figure 3:**
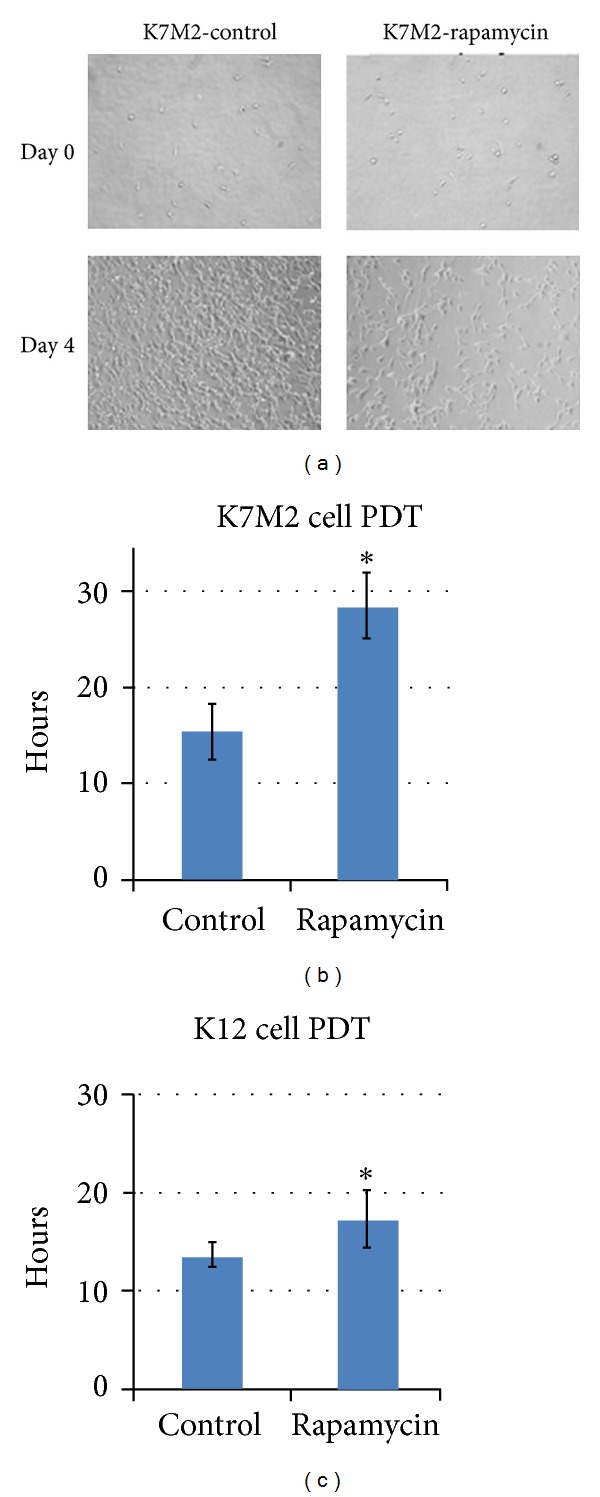
Rapamycin treatment reduces K7M2 cell proliferation. (a) K7M2 cells were cultured with media containing 10 *μ*M rapamycin or DMSO only (control) for four days. Representative images of cell density at the beginning and end of treatment are shown. (b) A quantitative analysis of (a) to determine the population doubling time (PDT) of K7M2 cells in rapamycin-treated and control groups (DMSO only). (c) A quantitative analysis determining the population doubling time (PDT) of K12 cells in rapamycin-treated and control groups (DMSO only). Asterisks (∗) indicate statistically significant differences (*P* < 0.05).

We analyzed K7M2 cell proliferation with and without rapamycin treatment because rapamycin has been shown to induce a G1-arrest in many tumor cell lines including osteosarcoma [10, 34]. After four days of culture in the presence or absence of rapamycin, K7M2 cells cultured in the presence of rapamycin appeared less dense on the culture dishes (Figure 3(a)). We then determined the population doubling time (PDT) of K7M2 and K12 cells with or without rapamycin treatment, over a culture period of 4 days (Figures 3(b) and 3(c)). It showed that PDT of both K7M2 and K12 was increased by rapamycin treatment, while its effect on K7M2 cells is more profound than that of K12 cells.

### 3.4. Rapamycin Treatment Reduces K7M2 Cell Migration and Invasion

**Figure 4:**
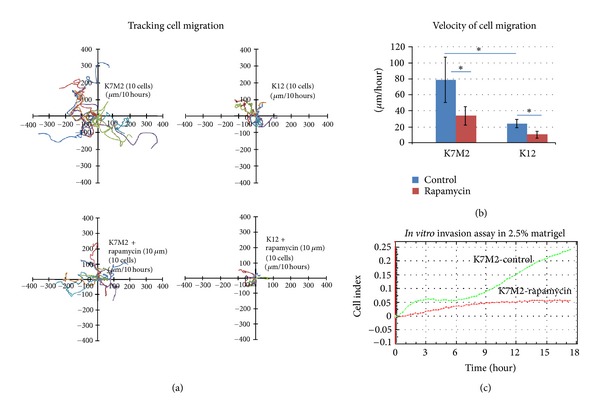
OS cell migration and invasion is sensitive to rapamycin treatment. (a) K7M2 and K12 cell migration was tracked in the presence or absence (DMSO only control) of rapamycin (10 *μ*M) and the data obtained is displayed. (b) The velocity of cell migration was determined in each group in (a) and the results are displayed. (c) K7M2 cell invasion through a 2.5% Matrigel was monitored for 18 hours in the presence or absence (DMSO only) of 10 *μ*M rapamycin. Asterisks (∗) indicate statistically significant differences (*P* < 0.05).

K7M2 cells display much greater metastatic potential *in vivo* compared to K12 cells [24]. Consistent with this enhanced metastatic ability, we performed an *in vitro* tracking cell migration assay and observed more migratory activity in K7M2 cells compared to K12 cells and both K7M2 and K12 migration was sensitive to rapamycin (Figure 4(a)). We then determined the velocity of cell migration in this assay and the results are similar and displayed in Figure 4(b). Lastly, we evaluated the invasion capacity of K7M2 and K12 cells in 2.5% Matrigel and found that K7M2 cells have much stronger invasion capacity than K12 cells (data not shown). Furthermore, it was found that rapamycin greatly reduces K7M2 cell invasion in this assay (Figure 4(c)).

### 3.5. ALDH-High K7M2 Cells Have Greater Invasiveness, Morphologic Changes, Resistance to Oxidative Stress, and Expression of Oncogenic Factors Than ALDH-Low K7M2 Cells

**Figure 5:**
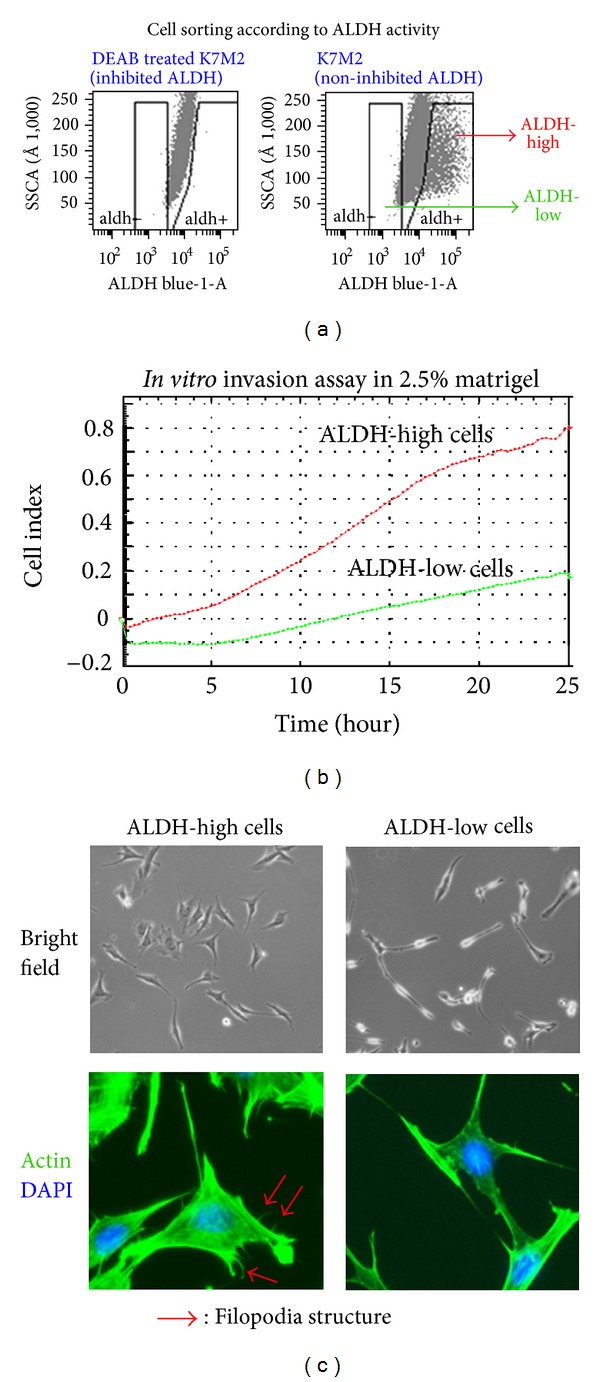
Cell sorting of K7M2 cells via ALDH activity and differential features of ALDH-high cells and ALDH-low cells. (a) K7M2 cells were suspended in Aldefluor buffer and sorted according to their fluorescence. Cells were treated with DEAB to block ALDH fluorescence, and cells were deemed ALDH-high if their fluorescence was higher than that of the DEAB-treated controls. Cells were deemed ALDH-low if their fluorescence was lower than that of the DEAB-treated controls. (b) ALDH-high and ALDH-low K7M2 cell invasion was tracked over a period of 24 hours and the data obtained is displayed. The ALDH-high cells displayed a much higher invasion potential, with more fraction of cells invading through the matrigel (2.5%). (c) Under bright field microscopy and under fluorescent microscopy (after staining for actin (green) and nucleic acids (blue)) ALDH-high K7M2 cells displayed more filopodia than ALDH-low cells. Also the ALDH-high cells were more irregularly shaped and were more pleomorphic than ALDH-low K7M2 cells.

K7M2 cells have been sorted according to the differential ALDH activity. The sorted populations of ALDH-high and ALDH-low K7M2 cells have been expanded *in vitro* (Figure 5(a)). *In vitro* invasion assay with 2.5% Matrigel demonstrated that ALDH-high K7M2 cells have much higher invasion capacity compared to ALDH-low K7M2 cells (Figure 5(b)). ALDH-high K7M2 cells were found to be more spread out and irregular in shape than ALDH-low K7M2 cells. They also displayed characteristics of typical motile behavior such as more organized structure of actin and the presence of filopodia (Figure 5(c)). Certain types of filopodia have been associated with increased invasiveness and metastasis rate [35]. Antioxidative stress assay showed that ALDH-high K7M2 cells are more resistant to the treatment of hydrogen peroxide, compared to ALDH-low K7M2 cells (Figure 2). RT-PCR also showed that ALDH-high cells had a higher expression of mTOR and c-Myc.

### 3.6. ALDH-High K7M2 Cells Treated with an ALDH-Inhibitor (Disulfiram) Show Reduced ALDH Activity, Oncogene Expression, and Morphologic Alterations

**Figure 6:**
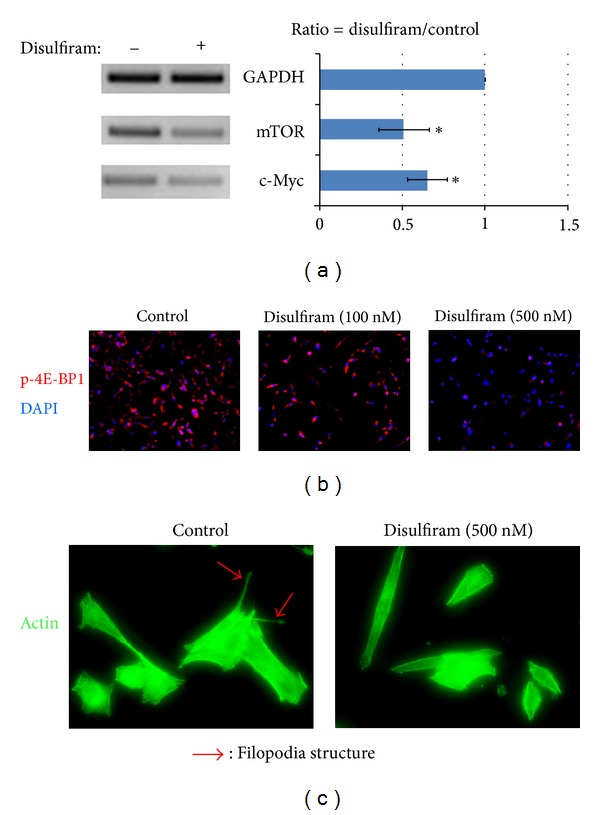
Inhibition of ALDH with disulfiram inhibits metastatic properties of ALDH-high K7M2 cells. (a) Disulfiram (250 nM) was added to ALDH-high K7M2 cells and the cells were cultured for at least 24 hours in 10% FBS growth medium. RT-PCR was used to determine gene expression differences as a result of this treatment, with GADPH used as a control. Both mTOR and c-Myc expression were reduced as a result of the treatment with disulfiram. (b) Immunostaining with phospho-4E-BP1 (a mouse anti-mTOR antibody) was done to confirm that treatment with disulfiram reduced expression of mTOR As the concentration of disulfiram was increased (0 nm, 100 nm and 500 nm) cells displayed progressively less mTOR expression. (c) Morphologic differences after treatment with disulfriam were also present, with disulfiram treated cells (stained for actin) appearing less pleomorphic and with fewer filopodia.

ALDH-high cells treated with disulfiram were verified to show a much reduced ALDH activity (data not shown). ALDH-high cells treated with disulfiram were found to have a lower expression of mTOR and c-Myc than untreated ALDH-high cells as shown via RT-PCR (Figure 6(a)). Treated cells also displayed less mTOR activity as shown via immunostaining with phosphpo-4E-BP1 (Figure 6(b)). Finally, the addition of disulfiram altered the cells' morphologies: they appeared much more like ALDH-low cells than untreated ALDH-high cells. There were much fewer filapodia, and the cells appeared much more uniform in shape (Figure 6(c)).

## 4. Discussion

K7M2 murine OS cells are highly metastatic to the lungs and were clonally derived from the less metastatic K12 OS cells [24]. Thus, the K7M2 and K12 cell lines are very similar genetically but differ significantly in their metastatic phenotypes and represent excellent tools for determining the critical biochemical pathways of OS metastasis. Here we report that, in comparison with K12 cells, K7M2 cells feature “rapamycin-sensitive” mTOR signaling which promotes cellular behaviors associated with metastasis, including higher ALDH activity, increased resistance to oxidative stress, proliferation, migration, invasion, and upregulation of BMP2 and VEGF expression. These results imply that mTORC1 activity may contribute to the enhanced metastatic potential of K7M2 cells. 

There are several limitations to this study not the least of which is that all of our experiments were performed *in vitro*. Additionally, all of our experiments, and therefore our conclusions, are based entirely on the study of two murine cell lines. Although the observations reported above are interesting, care must be taken not to overstate these data. Fortunately, the metastatic phenotypes of  K7M2 and K12 cells have been well-characterized in previous studies, including the effect of rapamycin treatment on K7M2 metastasis [24, 36]. We believe that our conclusions are sound, but future studies evaluating ALDH and mTOR activity in other OS cell lines and *in vivo* will be essential to validate these observations. 

 A role for mTOR signaling in osteosarcoma metastasis is slowly evolving. Human osteosarcoma tissue samples have stained positive for mTOR signaling and this has been correlated with surgical stage, metastasis pattern, and disease-free survival [23]. Our current understanding of the molecular biology remains limited. It has been shown in K7M2 cells that Ezrin (a plasma membrane to actin cytoskeleton linker protein) expression is upregulated compared to K12 cells, and that dynamic regulation of Ezrin activity via phosphorylation is required for metastasis to occur in a mouse xenograft model. It appears that Ezrin must be turned “on” and “off” in order to regulate cellular metabolism and protect K7M2 OS cells against stress after early arrival in the lungs. Furthermore, Ezrin, phosphatidylinositol-3-kinase (PI3K), and AKT are each associated with phosphorylation of S6K1 and 4E-BP1. Interestingly, Ezrin-associated phosphorylation of S6K1 and 4E-BP1 is sensitive to rapamycin, suggesting that these phosphorylation events depend on the mTORC1 kinase complex [25, 37].

 mTOR signaling can regulate cell motility through mTORC1 and mTORC2-dependent kinase activity. Here we show that rapamycin can inhibit K7M2 cell migration *in vitro*, presumably by blocking mTORC1 activity. The regulation of cell motility by mTORC1 is most likely to be the mechanism that influences cell migration. Both mTORC1/S6K1 and mTORC1/4E-BP1 pathways are known to regulate cell motility. S6K1 participates in the phosphorylation of the focal adhesion proteins and F-actin reorganization in order to modulate cell motility but little is known about the manner in which 4E-BP1 regulates this process [14]. Thus, rapamycin treatment could block migration by interfering with mTORC1-mediated cell motility but would potentially leave mTORC2 unchecked and free to regulate cell motility via control of the actin cytoskeleton through activation of the Rho GTPases [14]. 

ALDH activity has been identified as a “cancer stem cell” (CSC) marker in multiple neoplasms and has been associated with metastasis and a poor prognosis, but such a role has yet to be established in osteosarcoma [26–30]. The CSC hypothesis predicts that if certain genetic alterations occur in the right context, and within a more primitive cell, then a cancer-initiating cell is born that retains all the qualities of stem cells including self-renewal, proliferation, differentiation, resistance to drugs, stress, apoptosis, and the capacity to migrate, invade, and induce angiogenesis [38]. Our results show that ALDH-high OS cells display a significantly greater metastatic phenotype than ALDH-low cells. Although the relationship between CSCs and metastasis has not been clearly elucidated, it has been demonstrated that the number of metastatic cancer colonies correlates with the frequency of CSCs in the primary tissue. Furthermore, CSC subpopulations display a higher potential for invasiveness than subpopulations of nonstem tumor cells [39]. 

In this study, we observed that K7M2 cells exhibit higher levels of ALDH activity and this is dependent on mTORC1 activity as indicated by treatment with rapamycin. Furthermore, we showed that K7M2 cells, in comparison to K12 cells, are more resistant to oxidative stress caused by H_2_O_2_ treatment. The activity of ALDH in cancer may function to neutralize oxidative stress and provide chemoresistance, and thus upregulation of ALDH activity by mTOR may be one mechanism that confers resistance to oxidative stress in K7M2 cells [32, 33]. Alternatively, disulfiram, an ALDH inhibitor, has been shown to reduce the invasiveness of U2OS cells and the expression of matrix metalloproteinases, raising the possibility that ALDH activity may have a more direct role in tumor invasion [28]. However, the mechanisms for disulfiram's effects on U2OS invasion remain unclear.

In terms of stem cell regulation, an integral role for mTOR signaling in the proliferation and self-renewal of embryonic stem cells (ESCs) has been implicated. Loss of mTOR activity in human ESCs impairs pluripotency, blocks proliferation, and enhances mesoderm and endoderm activities [40]. The repression of developmental genes through mTOR signaling is thought to maintain pluripotency. Differentiation induces an “anabolic switch” and an increase in protein synthesis and protein content coincide with cell fate determination. Specifically, mTOR, and 4E-BP1 may function to regulate global and selective protein synthesis during self-renewal and differentiation [41]. Therefore, mTORC1 kinase activity may serve a similar role in the regulation of osteosarcoma stem cell proliferation and self-renewal. 

Indeed, the ability of mTOR to promote proliferation without differentiation has been demonstrated in the literature. Many studies have shown that rapamycin treatment induces a G1-arrest in various cell lines, including osteosarcoma [10, 34], and here we have shown that rapamycin has a similar effect on K7M2 OS cells. This G1-arrest is likely to occur through the down-regulation of cyclin D1 as was previously shown in human OS cells by Gazitt et al. [42]. mTOR activity has also been shown to block differentiation as rapamycin treatment can enhance osteoblast differentiation [43]. Although an OS cancer stem cell has not been identified to date, evidence is accumulating that suggests this discovery is merely a matter of time [44]. If an OS cancer stem cell exists, we suspect that mTOR and ALDH activity play pivotal roles in the maintenance of this subpopulation of cancer cells. 

 Here we also demonstrated that BMP2 and VEGF expression in K7M2 cells is reduced by rapamycin treatment. Previously we had shown that BMP2 and VEGF expression is upregulated in K7M2 cells compared to K12 cells [31]. Furthermore, treatment with the BMP antagonist Noggin caused decreased motility, altered morphology, and increased cell death in K7M2 cells. It is also known that BMP2 is activated through hypoxia/AKT/mTOR/HIF*α* pathway in osteoblasts [45]. VEGF, on the other hand, is a potent inducer of neovascularization whose activity is thought to be an absolute requirement for tumor growth and metastasis [46]. Consistent with the present study, rapamycin has been shown to reduce VEGF production and inhibit angiogenesis in mice *in vivo* [47]. Therefore, down-regulation of BMP2 and VEGF (by mTORC1 inhibition) may each contribute to alter the metastatic behavior of K7M2 cells. Both BMP2 and VEGF may prove to be therapeutic targets in the treatment of OS metastasis. *In vivo* studies are essential to investigate the role of these secreted proteins in OS metastatic biology.

Our results show that ALDH inhibition via disulfiram actually decreased mTOR activity. This implies that under certain circumstances, mTOR activity may be regulated by ALDH. This has profound implications as mTOR activity is important in many cancers and is downstream of important tumor suppressors and oncogenes that are frequently mutated in human cancers [17]. Our work supports an oncogenic role for mTORC1 activity in osteosarcoma and provides evidence to link mTOR signaling, ALDH activity, and metastatic behavior. To our knowledge the present study is the first to implicate mTOR in the regulation of ALDH activity. Given our data and other evidence that exists to implicate mTOR and ALDH function in CSCs, we suspect that if an OS stem cell exists, mTOR and ALDH may be important for its proliferation, self-renewal, drug resistance, cell survival, and metastasis. The search for surface markers of OS cells has been long and disappointing, but ALDH may represent great promise as an OS stem cell surface marker. At this point in time we cannot be certain of the mechanisms by which ALDH and mTOR interact. Future investigations are required to further delineate the roles and relationships of mTOR, ALDH, and OS cancer stem cells. 

In conclusion, mTOR signaling is upregulated in highly metastatic K7M2 OS cells. This upregulation is associated with increased ALDH expression and activity, resistance to oxidative stress, greater proliferation, migration, and invasion, and higher BMP2 and VEGF expression. This work implicates rapamycin and its analogs as potential agents in the treatment and prevention of OS metastasis. It also suggests a relationship between mTOR and ALDH. ALDH may, in its own right, represent a possible marker and therapeutic target of OS cell metastatic potential.

## Figures and Tables

**Table 1:** Primer Sequences.

Gene	Primer sequence	Band size (bp)
GAPDH	Forward: TCCATGACAACTTTGGCATTG	103
	Reverse: TCACGCCACAGCTTTCCA	
BMP2	Forward: TCTTCCGGGAACAGATACAGG	126
	Reverse: TGGTGTCCAATAGTCTGGTCA	
BMP4	Forward: ATTCCTGGTAACCGAATGCTG	89
	Reverse: CCGGTCTCAGGTATCAAACTAGC	
VEGF	Forward: GCCAGACAGGGTTGCCATAC	108
	Reverse: GGAGTGGGATGGATGATGTCAG	
c-Myc	Forward: TGACCTAACTCGAGGAGGAGCTGGAATC	170
	Reverse: AAGTTTGAGGCAGTTAAAATTATGGCTGAAGC	
ALDH	Forward: GACAGGCTTTCCAGATTGGCTC	142
	Reverse: AAGACTTTCCCACCATTGAGTGC	
mTOR	Forward: CAGTTCGCCAGTGGACTGAAG	130
	Reverse: GCTGGTCATAGAAGCGAGTAGAC	
